# A cross-attentive multi-task graph learning framework for chemical reaction modeling

**DOI:** 10.1093/bioinformatics/btag193

**Published:** 2026-04-20

**Authors:** Maryam Astero, Anchen Li, Elena Casiraghi, Juho Rousu

**Affiliations:** Department of Computer Science, Aalto University, Espoo 02150, Finland; Department of Computer Science, Aalto University, Espoo 02150, Finland; Department of Computer Science, Aalto University, Espoo 02150, Finland; Department of Computer Science, Università degli Studi di Milano, Milan 20122, Italy; Department of Computer Science, Aalto University, Espoo 02150, Finland; Department of Computer Science, University of Helsinki, Helsinki 00014, Finland

## Abstract

**Motivation:**

Understanding chemical reactions requires bridging fine-grained molecular edits with broader semantic context. Reaction mechanisms are determined not only by local atom–bond transformations but also by the global reaction class. However, most existing approaches treat these tasks separately or rely on external atom-mapping tools, introducing noise and limiting end-to-end learnability. We introduce MARCC (Mapping-Assisted Reaction Center and Classification), a multi-task graph neural network that jointly predicts atom mappings, reaction centers, and reaction classes within a unified architecture.

**Results:**

MARCC integrates three key innovations: (i) a mapping-guided cross-attention mechanism that aligns reactants and products for local edit detection, (ii) a dual-graph design that explicitly reasons about bond-level transformations, and (iii) pooled product embeddings for global reaction classification. On the USPTO-50K benchmark, MARCC achieves state-of-the-art results when trained with both reactants and products, including 98.2% atom mapping accuracy, 99.1% Top-1 edit localization accuracy, and 97.2% reaction classification accuracy. Even under the products-only setting, MARCC delivers competitive performance comparable to specialized baselines. Ablation studies confirm the value of mapping-guided attention and multi-task supervision, which enhance both predictive accuracy and interpretability. By unifying atom-level alignment, local reactivity, and global classification, MARCC provides a structured and interpretable framework for reaction understanding. Beyond benchmarks, MARCC has the potential to support applications in reaction annotation, template discovery, and mechanism inference; with additional domain-specific modeling and data, it could be extended to biochemical domains such as enzyme-catalyzed transformations and metabolic pathway modeling.

**Availability and implementation:**

The source code and implementation details are available at https://github.com/maryamastero/MARCC and archived at https://doi.org/10.5281/zenodo.18500230.

## Introduction

Understanding chemical reactions requires reasoning across multiple structural and semantic levels. Three core tasks underpin this process: *atom mapping*, which establishes atom-level correspondences between reactants and products; *reaction center identification*, which pinpoints the atoms and bonds altered during the transformation; and *reaction classification*, which captures the overall transformation type. Together, these tasks provide complementary perspectives on molecular reactivity and are essential for applications where both reaction sides are available, including mechanism inference, reaction template discovery, role assignment, and reaction annotation, as well as supervised training settings for retrosynthesis.

Atom mapping, traditionally solved by graph-theoretic search (Raymond and Willett 2002, Heinonen et al. 2011), is now increasingly addressed by attention- or graph-based methods (Schwaller et al. 2021, Nugmanov et al. 2022, Astero and Rousu, 2024, 2025). Reaction center prediction initially relied on rule-based or template-driven systems (Coley et al. 2017, Dai et al. 2019), later reformulated as graph-labeling or subgraph selection tasks (Yan et al. 2020, Somnath et al. 2021, Lan et al. 2024). Reaction classification has similarly evolved from fingerprint-based methods (Gelernter et al. 1990, Schneider et al. 2015, Probst et al. 2022) to graph neural network models (Li et al. 2024). Despite these advances, most approaches treat these tasks in isolation, overlooking their interdependence: atom mappings provide a structural scaffold for localizing reactive centers, and both local edits and mappings jointly constrain global reaction classes.

Prior research on atom mapping, such as *SAMMNet* (Astero and Rousu 2025), demonstrated that multitask learning can partially mitigate the inherent incompleteness of reaction data by coupling atom mapping with auxiliary supervision. Specifically, SAMMNet focuses primarily on correspondence prediction and incorporates a self-supervised auxiliary task of atom-type prediction to improve generalization and robustness in situations with incomplete reaction information. However, it did not explicitly unify local reaction mechanisms with global reaction semantics, nor did it model bond-level transformations or reaction-level abstractions within a single framework.

We introduce MARCC (Mapping-Assisted Reaction Center and Classification), a cross-attentive multi-task framework that explicitly unifies atom mapping, reaction center identification, and reaction classification within a single learning architecture. MARCC leverages mapping-guided cross-attention to align reactant and product graphs, enabling joint reasoning over local structural changes and global reaction semantics. Furthermore, a dual-graph representation treats bonds as explicit nodes, allowing the model to directly capture bond-breaking and bond-forming events that define reaction mechanisms.

This unified design enables MARCC to jointly reason over atom-mapping prediction, mechanistic edits, and reaction semantics, rather than treating these components as independent prediction problems.

Evaluated on the USPTO-50K benchmark, MARCC delivers state-of-the-art performance in reaction center identification and classification, while achieving mapping accuracy on par with specialized systems. These results establish MARCC as a powerful and interpretable framework for chemical reaction understanding. Beyond chemical informatics, the approach has natural extensions to biological settings: many biochemical processes, such as enzyme-catalyzed transformations, metabolic pathway progression, and drug metabolism, can be represented as graph-structured reactions with localized centers and global functional classes. By jointly learning atom-level correspondences, reactive sites, and overall reaction types, MARCC offers a structured foundation for applications in systems biology, enzymology, and pharmacokinetics.

Our key contributions are as follows:

- We propose a unified multi-task GNN architecture that simultaneously performs reaction classification, reaction center prediction, and atom mapping within a single framework.
- We introduce a cross-graph attention mechanism that explicitly aligns reactant and product atoms, enabling joint inference across molecular structures.
- We design a dual-graph representation in which bonds are treated as explicit nodes, allowing the model to capture bond-level transformations more effectively.
- We develop an imbalance-aware optimization scheme that combines focal and dice losses to improve robustness on underrepresented reaction classes and rare transformation patterns.

To ensure fair comparison with prior reaction-center baselines, which typically operate in a retrosynthesis setting using only product graphs, we additionally evaluate a *products-only* variant of MARCC. In this setting, the cross-attention module is disabled for reactivity prediction and used solely for classification, aligning MARCC with the input constraints of existing baselines.

## Problem formulation

We formulate chemical reaction modeling as a multi-task learning problem over molecular graph transformations. Each reaction instance is represented by a pair of molecular graphs: the major product $G_{P}$ and its corresponding reactants $G_{R}$, where atoms are nodes and bonds are edges.

We define a molecular graph as G=(V,A,X,E), where *V* is the set of atoms, $A\in {\left\{0,1\right\}}^{\left|V|\times |V\right|}$ is the adjacency matrix, $X\in R^{|V|\times d_{x}}$ encodes atom features (e.g. element, charge, hybridization), and $E\in R^{|E|\times d_{e}}$ encodes bond features (e.g. bond type, conjugation, ring membership). Notably, *G* is not required to be a single connected component; it may represent a set of multiple molecules (e.g. reactants and reagents), allowing the model to represent complex multi-component reaction systems without structural modification.

Given a reaction $\left(G_{P},G_{R}\right)$, our goal is to jointly model two structurally coupled tasks—*reaction center identification* and *reaction classification*—while leveraging *atom mapping* as an auxiliary alignment signal.

### Atom mapping

Atom mapping establishes a one-to-one correspondence between atoms in the reactants and those in the product, capturing transformation mechanics at atomic resolution. This information directly informs both center prediction and reaction class classification.

In practice, patent-derived data often include side products or incomplete reactant specification, making direct mappings ambiguous. To mitigate this, we reverse the mapping direction: each product atom is softly aligned to candidate reactant counterparts, analogous to retrosynthetic reasoning.

We represent this alignment as a differentiable correspondence matrix: $M\in {\left[0,1\right]}^{\left|V_{P}|\times |V_{R}\right|},\;M_{i,i^{\prime }}=Pr(v_{i}^{P}↔v_{i^{\prime }}^{R}),$ where $M_{i,i^{\prime }}$ denotes the probability that product atom $v_{i}^{P}$ aligns with reactant atom $v_{i^{\prime }}^{R}$. The matrix *M* is constrained to be doubly stochastic, i.e. each row and column sums to one, ensuring a soft one-to-one correspondence.

Atom mappings are optimized by maximizing structural consistency:


$$M^{*}=\arg\underset{M}{\max}\sum_{i,j}\sum_{i^{\prime },j^{\prime }}A_{P}(i,j)\cdot A_{R}(i^{\prime },j^{\prime })\cdot M_{i,i^{\prime }}\cdot M_{j,j^{\prime }}.$$

### Reaction center identification

The reaction center is the subset of atoms and bonds in $G_{P}$ that undergo structural changes relative to their aligned counterparts in $G_{R}$. These changes include bond formation/cleavage, bond order modifications, or alterations in atomic valence, hydrogen count, or charge.

We model center detection as binary classification on the product graph. Each atom $v_{i}^{P}\in V_{P}$ and bond $e_{ij}^{P}\in E_{P}$ is assigned binary labels: $y_{i}^{\text{atom}}\in \{0,1\},\;z_{ij}^{\text{bond}}\in \{0,1\},$ where a label of 1 indicates structural reactivity. Atom mappings provide the alignment needed for pairwise comparison between $G_{P}$ and $G_{R}$.

### Reaction classification

At a global level, each reaction is assigned to one of *K* classes (e.g. substitutions, eliminations, additions) based on transformation semantics. We model this as multi-class prediction: $c^{*}=\text{arg}{\text{max}}_{k\in \{1,\ldots ,K\}}\mathrm{Pr}(c=k|G_{P},G_{R}),$ where *c* denotes the predicted reaction class.

## Mapping-assisted reaction center and classification

**Figure 1:**
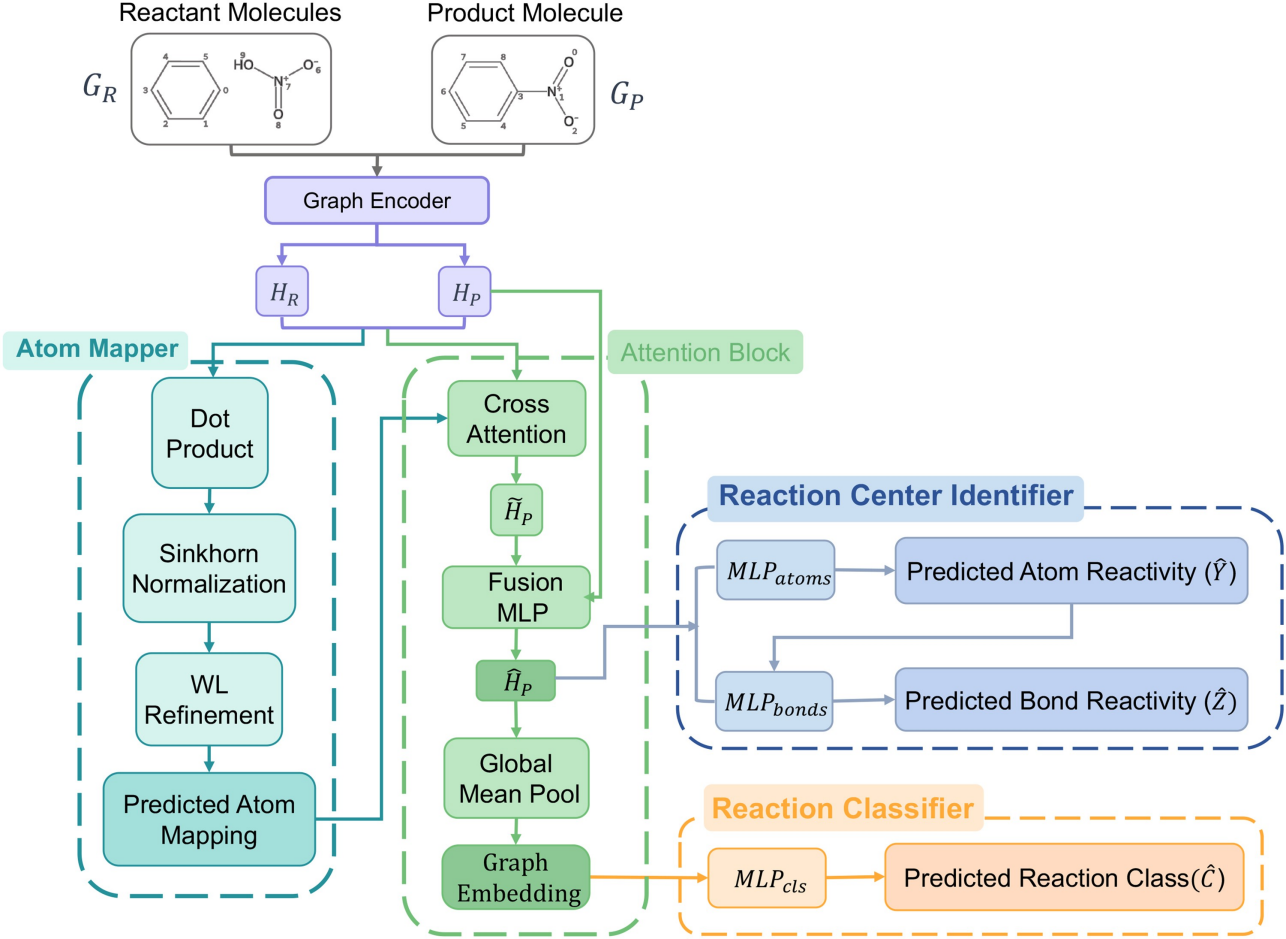
Overview of the MARCC architecture. Reactant ($G_{R}$) and product ($G_{P}$) graphs are encoded by a shared GINE encoder, producing node embeddings $H_{R}$ and $H_{P}$. The Atom Mapper computes soft alignments via dot-product similarity and Sinkhorn normalization, refined by Weisfeiler–Lehman (WL) symmetry resolution. These correspondences guide a cross-attention mechanism, yielding enriched product embeddings ${\hat{H}}_{P}$. Task-specific heads then perform Reaction Center Identification (atom/bond reactivity) and Reaction Classification (global reaction type).

We propose MARCC (Mapping-Assisted Reaction Center and Classification), a multi-task graph neural architecture for joint atom mapping, reaction center prediction, and reaction classification. The tasks are coupled through a shared embedding space and mapping-guided attention (Fig. 1). A detailed algorithm is given in Supplementary Material S1.

### Graph encoder

Reactant and product graphs are independently encoded by a shared GINE encoder (Fey and Lenssen, 2019) with residual connections: $H_{R}=\text{GINE}(A_{R},X_{R},E_{R}),\;H_{P}=\text{GINE}(A_{P},X_{P},E_{P}),$ where $H_{R}$ and $H_{P}$ denote node embeddings.

### Atom mapping

Atom correspondences are estimated by soft graph matching. A raw similarity matrix is computed as: $\hat{M}=H_{P}H_{R}^{⊤},$ then normalized by Sinkhorn scaling (Sinkhorn and Knopp 1967) into a doubly stochastic matrix *M*. Final mappings are obtained by row-wise softmax, aligning each product atom to a reactant candidate.

#### Symmetry refinement

To improve the quality of atom mapping predictions, especially in cases involving structural symmetries (e.g. aromatic rings or repetitive substructures), we apply a refinement procedure based on the Weisfeiler–Lehman (WL) test (Weisfeiler and Leman 1968). The WL algorithm iteratively updates atomic labels via neighborhood aggregation. Atoms with identical labels across all iterations and matching atomic types are grouped into equivalence classes. These classes are used to disambiguate alignments during both training and inference, following (Astero and Rousu 2025).

### Mapping-guided cross-attention

To connect mappings with downstream tasks, each product atom attends only to its most probable reactant counterpart:


$$q_{i}=W_{Q}H_{P}^{\left(i\right)},\;k_{i}=W_{K}H_{R}^{\left(M_{i}\right)},\;v_{i}=W_{V}H_{R}^{\left(M_{i}\right)},$$

$${\tilde{H}}_{P}^{\left(i\right)}=\text{softmax}\left(\frac{q_{i}^{⊤}k_{i}}{\sqrt{d_{k}}}\right)v_{i},$$

with trainable weights $W_{Q},W_{K},W_{V}$. Final embeddings are obtained by concatenation: ${\hat{H}}_{P}^{\left(i\right)}=[H_{P}^{\left(i\right)}||{\tilde{H}}_{P}^{\left(i\right)}]$, where || denotes feature-wise concatenation. Ground-truth mappings supervise training, while predicted mappings are used at inference.

### Reaction center identification

Atom and bond reactivity are modeled as binary classification tasks on the product graph. Atom logits are: ${\hat{y}}_{i}=\sigma \left({\text{MLP}}_{\text{atom}}({\hat{H}}_{P}^{\left(i\right)})\right).$

Bond predictions combine atom embeddings, bond features, dual-graph features, and reactivity logits:


$${\hat{z}}_{ij}=\sigma \left({\text{MLP}}_{\text{bond}}([{\hat{H}}_{P}^{\left(i\right)}||{\hat{H}}_{P}^{\left(j\right)}||E_{P}^{\left(ij\right)}||H_{D}^{ij}||{\hat{y}}_{i}||{\hat{y}}_{j}])\right).$$

#### Dual graph

To capture bond-centric context, each bond is treated as a node in a dual graph, with edges connecting bonds that share an atom. Bond features form node inputs, while connectivity is inherited from shared atoms (see Supplementary Material S2).

### Reaction classification

Global semantics are captured by mean-pooling product embeddings: ${\bar{H}}_{P}=\frac{1}{\left|V_{P}\right|}\sum_{i\in V_{P}}{\hat{H}}_{P}^{\left(i\right)},\;\hat{c}=\text{softmax}({\text{MLP}}_{\text{class}}({\bar{H}}_{P})).$

### Multi-task objective

The overall loss is:


$$L_{\text{total}}=\lambda_{\text{map}}L_{\text{map}}+\lambda_{\text{react}}(L_{\text{atom}}+L_{\text{bond}})+\lambda_{\text{cls}}L_{\text{cls}}.$$

#### Reactivity loss

Because reactive atoms/bonds are sparse, we combine focal loss (Lin et al. 2017) (to emphasize hard cases) with Dice loss (Milletari et al. 2016) (to optimize overlap):


$$\begin{array}{cc} L_{\text{atom}} & =\lambda_{\text{dice}}L_{\text{Dice}}^{\text{atom}}+(1-\lambda_{\text{dice}})L_{\text{Focal}}^{\text{atom}},\\ L_{\text{bond}} & =\lambda_{\text{dice}}L_{\text{Dice}}^{\text{bond}}+(1-\lambda_{\text{dice}})L_{\text{Focal}}^{\text{bond}}. \end{array}$$

The full mathematical definitions of the Dice and focal loss terms are given in Supplementary Material S3.

## Experiments

### Experimental setup

#### Dataset

We evaluate MARCC on the USPTO-50K benchmark dataset (Lowe 2012, Schneider et al. 2016), using the standardized, atom-mapped release from Somnath et al. (2021). The dataset consists of 50,000 reactions annotated with atom mappings and categorized into 10 reaction classes, making it a widely adopted benchmark for reaction understanding.

To mitigate spurious correlations arising from atom index ordering, we adopt the canonicalization and remapping procedure of (Somnath et al. 2021; Astero and Rousu 2024), which uses canonical product SMILES and regenerated alignments. This ensures that learned signals reflect true structural reasoning rather than positional artifacts.

Atom and bond reactivity labels are derived by comparing reactants and products: atoms are labeled reactive if their hydrogen count or formal charge changes; bonds are labeled reactive if broken or altered in type. As only 0.5% of bonds are newly formed, we focus on transformations over existing bonds, consistent with prior work (Somnath et al. 2021). Following the standard protocol, we adopt an 8:1:1 training, validation, and test split.

We prioritize the USPTO-50K benchmark over larger, uncurated corpora (e.g. USPTO-full) because its high-fidelity ground truth is essential for isolating the architectural contributions of our multi-task framework. This ensures that performance metrics reflect the model’s structural reasoning rather than the noise or erroneous mappings prevalent in larger datasets.

#### Graph construction and features

Molecular graphs are constructed from SMILES representations using RDKit (Landrum et al. 2006). Atom features include atomic number, formal charge, chirality, hybridization, aromaticity, degree, hydrogen counts, and ring membership. Bond features capture bond type, conjugation, stereochemistry, and ring membership. All features are one-hot encoded or categorical and concatenated into atom and bond embeddings. A full specification is provided in Material S4.

#### Evaluation metrics

We assess performance on reaction center prediction, reaction classification, and atom mapping.

- **Reaction Center Identification**: evaluated using Top-*n* Edit Accuracy (n∈{1,3,5}), which measures the proportion of true edits recovered among the top-ranked predictions.
- **Reaction Classification**: evaluated using Top-1 Accuracy, reflecting the proportion of reactions assigned to the correct class.
- **Atom Mapping**: evaluated using symmetry-aware accuracy (Astero and Rousu 2025), which accounts for equivalence among symmetrically valid mappings.

#### Hyperparameter search

We optimized hyperparameters using Optuna’s Tree-structured Parzen Estimator (TPE) sampler (Bergstra et al. 2011). The search space covered architectural choices (embedding dimension, encoder depth, number of attention heads), optimizer settings, task weights, and loss coefficients. Continuous parameters (e.g. learning rate) were sampled on a logarithmic scale, while discrete and categorical options were sampled directly.

Hyperparameter selection was performed exclusively on the validation set. After identifying the best configuration, we retrained the model from scratch on the combined training and validation sets and reported results on the held-out test set.

Each trial was scored using a scalarized validation objective that jointly balances atom-level F1, bond-level F1, and reaction classification F1. To improve efficiency, underperforming trials were pruned early using Optuna’s pruning mechanisms—primarily the Asynchronous Successive Halving (ASHA) strategy, with the Median Pruner used as a fallback (Akiba et al. 2019).

For every model–mode combination, we conducted 30 trials per study. The best-performing configuration was reused across all reported experiments to ensure comparability. Details of the search space, trial budget, and pruning strategies are provided in Supplementary Material S5.

#### Task weighting

We adopt uncertainty-based weighting (Kendall et al. 2018) to balance the three tasks. Each task is assigned a learnable log-variance parameter, leading to the joint loss: $L_{\text{total}}=\sum_{i}\left(\frac{1}{2\sigma_{i}^{2}}L_{{\text{task}}_{i}}+\text{log}\;\sigma_{i}\right),$ where $\sigma_{i}$ denotes the homoscedastic uncertainty of task *i*. A larger $\sigma_{i}$ downweights the corresponding loss, while a smaller $\sigma_{i}$ increases its influence. This scheme adaptively rescales gradients, giving more weight to reliable tasks and reducing the impact of noisier ones. The uncertainty parameters $\sigma_{i}$ are learned jointly with the model weights during training and remain fixed at inference.

#### Training setup

All experiments were conducted on the Aalto University Triton HPC cluster using a single NVIDIA V100 GPU (32 GB memory) with PyTorch Geometric. A typical MARCC training run on the USPTO-50K dataset, with batch size 32, 5 GINEConv layers (512-dimensional embeddings), 8 attention heads, and the AdamW optimizer (learning rate $1\times 10^{-4}$, weight decay $5\times 10^{-5}$), required approximately 17 hours of wall-clock time. Early stopping with a patience of 10 epochs was applied, and models typically converged within 45–50 epochs.

The shared graph encoder consists of 5 GINEConv layers with dynamically weighted skip connections. The guided cross-attention module employs 8 heads and integrates atom mapping alignments. Reactivity prediction heads were optimized with a hybrid Dice–Focal loss ($\lambda_{\text{dice}}=0.4$), with positive samples upweighted to address class imbalance. Thresholds for atom and bond reactivity were selected to maximize validation F1. Reaction classification was trained with standard cross-entropy loss across the 10 USPTO classes.

### Overall results and baseline comparison

We first evaluate MARCC on reaction center identification and reaction classification, comparing against strong baselines designed for retrosynthesis and reaction understanding. These include center-prediction methods [GraphRetro (Somnath et al. 2021), RetroXpert (Yan et al. 2020), RCSearcher (Lan et al. 2024)) and classification baselines (MolBERT (Fabian et al. 2020), RXGL (Li et al. 2024)]. Results are summarized in Table 1.

**Table 1:** Comparison with prior methods on USPTO-50K.

Model	**Reaction center: edit (%)**			Reaction classification (%)
	Top-1	Top-3	Top-5	Accuracy
GraphRetro	70.8	89.5	92.7	–
RetroXpert	50.4	61.1	62.3	–
RCSearcher	69.3	79.3	85.7	–
MolBERT	–	–	–	70.3
RXGL	–	–	–	93.2
**MARCC (Products Only)**	67.9	86.5	93.2	**97.6**
**MARCC (Full)**	**99.1**	**99.6**	**99.7**	**97.2**

“–” indicates the method does not perform that task. Best results in bold.

A key consideration is that most reaction-center baselines are designed for retrosynthesis and operate solely on the product graph, without leveraging reactant information. To provide a fair comparison, we also report a *products-only* variant of MARCC, in which the model is restricted to the major product structure. In this configuration, cross-attention is disabled for reactivity prediction and used only for classification.

As shown in Table 1, MARCC in the full-reaction setting achieves a Top-1 edit accuracy of 99.1%, far exceeding product-only baselines such as GraphRetro (70.8%). Since GraphRetro and related approaches operate solely on product graphs, the improvement reflects both MARCC’s richer input setting—leveraging explicit reactant–product alignment—and its integrated multi-task design. These gains are driven by three innovations: (i) multi-task supervision that couples local edits with global semantics; (ii) differentiable atom–atom alignment that resolves symmetries; and (iii) mapping-guided cross-attention that contextualizes node embeddings.

In the *products-only* setting, MARCC achieves a Top-1 edit accuracy of 67.9%. While this is comparable to product-only baselines, it highlights the intrinsic need for reactant–product alignment to reliably identify reactive atoms. Notably, classification accuracy remains very high (97.6%), surpassing specialized classifiers such as RXGL (93.2%) and MolBERT (70.3%). This suggests that while structural alignment is essential for fine-grained mechanistic inference, global reaction semantics can often be inferred directly from product structures alone.

Beyond reaction center prediction and classification, MARCC also learns atom-to-atom correspondences as part of its multitask training. In the following section, we evaluate its mapping accuracy and show that, despite not being the primary training objective, MARCC remains competitive with state-of-the-art atom mapping methods.

### Atom mapping evaluation

Atom-to-atom alignment has been extensively studied, with several methods designed specifically for this task. To contextualize the performance of MARCC’s auxiliary mapping head, we evaluate its accuracy on USPTO-50K against representative baselines: RXNMapper (Schwaller et al. 2021), GraphormerMapper (Nugmanov et al. 2022), and SAMMNet (Astero and Rousu 2025). Table 2 summarizes accuracies across these systems.

**Table 2:** Atom mapping accuracy on USPTO-50K.

Model	Accuracy (%)
RXNMapper	98.8
SAMMNet	97.4
GraphormerMapper	94.5
MARCC	98.2

In the full multitask setting, MARCC achieves 98.2% symmetry-aware accuracy, closely approaching RXNMapper (98.8%) while surpassing other graph-based models. Importantly, this performance is obtained without dedicating the architecture solely to atom mapping: the mapping head serves as a structural regularizer within the joint training scheme. This demonstrates that accurate atom alignments can be learned as a byproduct of multitask supervision, while simultaneously enhancing reaction center and classification performance (see Section Ablation: Impact of Multitask Learning for further analysis).

### Ablation: impact of multitask learning

To assess the contribution of each supervision signal, we ablate the MARCC training objective across seven task configurations (Table 3). Each configuration activates a subset of the available losses: reaction classification (Classification), reaction center prediction (Reactivity), and atom mapping (Mapping).

**Table 3:** Effect of supervision configurations on MARCC performance. Metrics include Top-1 Edit Accuracy, Reaction Classification Accuracy, and Mapping Accuracy.

Configuration	Edit Acc. (%)	Class Acc. (%)	Map Acc. (%)
Classification	–	73.3	–
Reactivity	87.7	–	–
Mapping	–	–	**99.0**
Classification + Reactivity	88.1	96.3	–
Mapping + Reactivity	93.0	–	90.5
Mapping + Classification	–	91.8	97.6
**Classification + Reactivity + Mapping (Full)**	**99.1**	**97.2**	98.2

“–” indicates the task is not applicable. Best values in bold.

Single-task training yields narrow competence: reaction classification alone (Classification) generalizes poorly (73.3%), while reaction-center (Reactivity) and Mapping models achieve decent accuracy but lack broader contextualization. Pairwise combinations provide complementary benefits—e.g. (Classification + Reactivity) substantially improves both classification (96.3%) and edit localization (88.1%), indicating strong synergy between global semantics and local edits.

Notably, mapping-only training achieves near-perfect alignment (99.0%) yet offers little transfer, reflecting overfitting to structural correspondences. By contrast, integrating mapping with center prediction (Mapping + Reactivity) or classification (Mapping + Classification) yields clear mutual reinforcement, raising edit and classification accuracy by 5–20 points.

The full multitask model achieves the best balance across objectives: 99.1% edit accuracy, 97.2% classification, and 98.2% mapping. This suggests that atom mapping acts as a structural regularizer, center prediction sharpens local reactivity, and classification enforces global semantic consistency—together yielding robust, generalizable representations.

Furthermore, we investigate the specific impact of the dual-graph (bond-node) representation. As detailed in Supplementary Material S9, removing the dual-graph module while retaining guided cross-attention results in a significant decline in edit accuracy, dropping from 99.1% to 85.4%. This confirms that modeling bonds as first-class nodes is essential for precisely localizing chemical transformations, a task that standard atom-centric message passing fails to capture with the same fidelity.

### Per-class performance analysis

**Figure 2:**
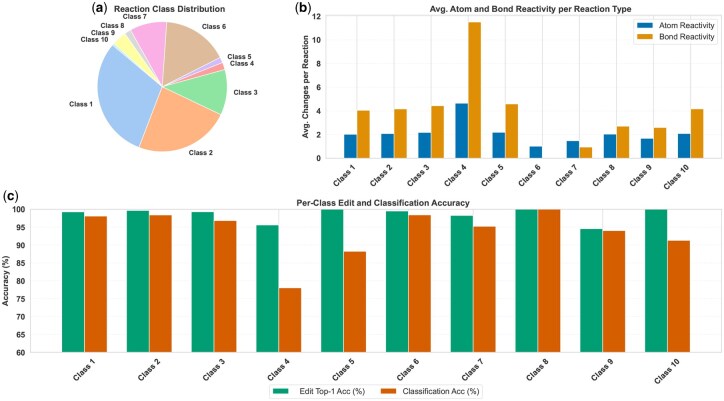
Dataset Composition and Per-Class Performance of MARCC on USPTO-50K. (a) Distribution of reaction classes in the test set, showing strong imbalance across the 10 categories. (b) Average number of reactive atoms and bonds per reaction type. (c) Per-class Top-1 edit localization accuracy and reaction classification accuracy achieved by MARCC.

To assess how structural and distributional factors affect predictive performance, we analyze per-class statistics including class frequency, reactivity profiles, and MARCC’s accuracy. Figure 2 summarizes MARCC’s performance across reaction classes. The dataset is highly imbalanced, with Classes 1 and 2 dominating while Classes 8–10 are underrepresented, limiting generalization. Reactivity profiles vary: Class 4 shows the most complex edits, whereas Classes 6 and 9 exhibit minimal transformations. Despite this variability, MARCC achieves consistently high edit localization accuracy across all classes, even for complex or sparse categories. In contrast, reaction classification accuracy is more affected by class imbalance, with weaker performance on rare classes (4, 5, 10). Importantly, prediction consistency analysis confirms that correct global classifications are strongly supported by accurate local edit predictions.

We further assess the internal consistency of MARCC’s predictions by measuring the proportion of reactions where both the reaction classification and Top-1 edit prediction are correct. As shown in Supplementary Material S6, this alignment remains consistently high across classes, suggesting that global predictions are supported by accurate local mechanistic edits.

### Case study: attention-guided interpretation

**Figure 3:**
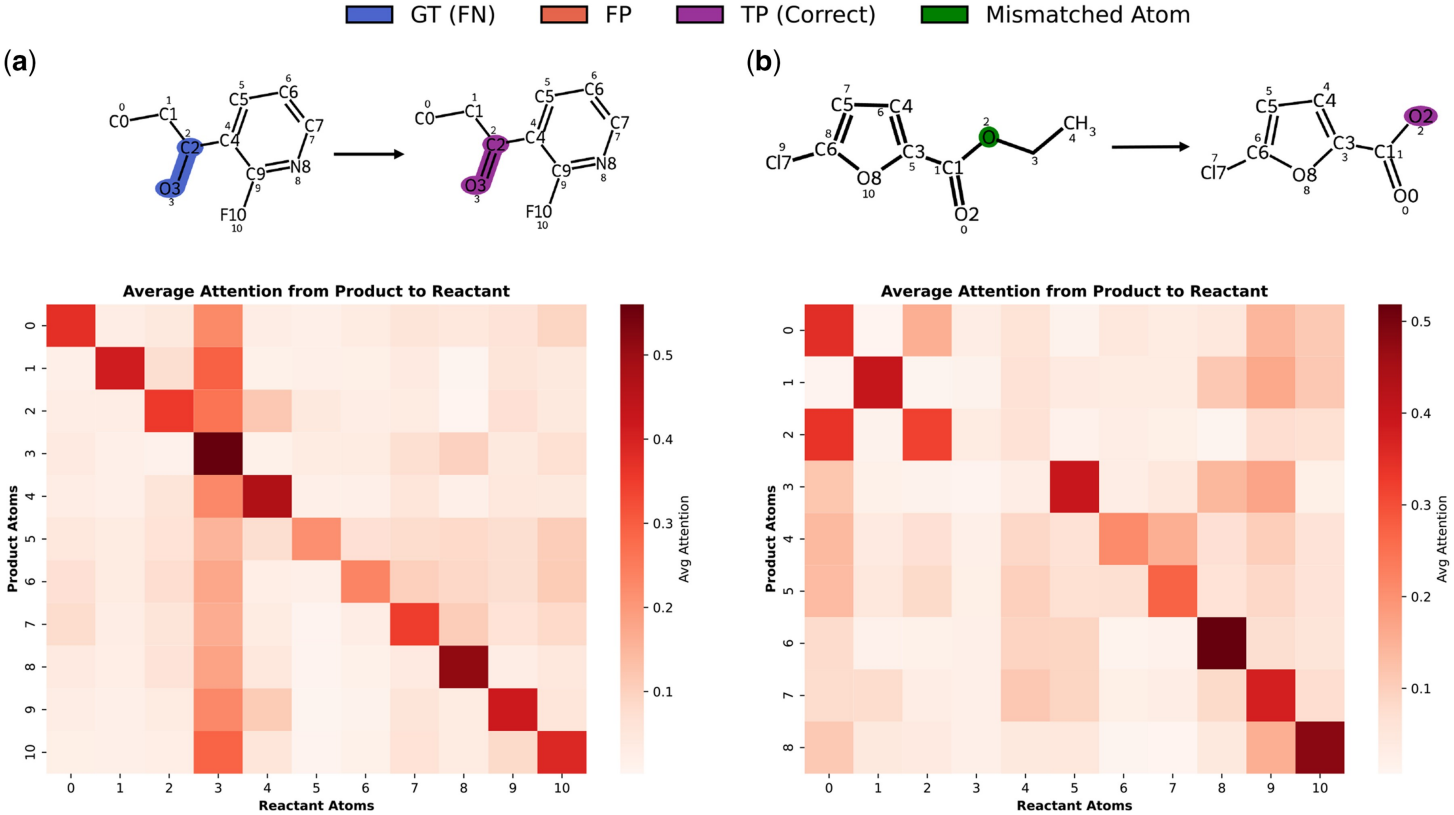
Visualization of attention-based alignment for interpretability and error analysis. Each panel shows one reaction example: the top row depicts the reaction with atom-level annotations, and the bottom row shows the corresponding attention heatmap from product atoms (y-axis) to reactant atoms (x-axis). (a) Correct prediction where both atom mapping and reaction center identification are accurate; attention weights concentrate on the true reactive atoms. (b) Partially misaligned case where atom mapping is imperfect due to symmetry in the product. Despite this, the model correctly localizes the reaction center, with attention focused on transformation-relevant atoms (e.g. oxygen), illustrating robustness to alignment noise. *Color coding*: Blue = ground-truth reactive atoms (FN), Orange = predicted reactive atoms not in ground truth (FP), Purple = correctly predicted reactive atoms (TP), Green = mismatched mapped atoms. *Heatmap*: Dark red = high attention, white = low or no attention.

To further assess the interpretability of MARCC and understand the interaction between atom mapping and reaction center prediction, we present qualitative visualizations of two representative examples. Figure 3 illustrates how MARCC’s cross-attention mechanism supports interpretability. In successful cases, attention weights align strongly with true reactive atoms, confirming that atom mapping and reaction center identification are correctly recovered. Even in challenging scenarios with partial symmetry, the model remains robust: despite imperfect mappings, attention focuses on chemically relevant atoms (e.g. oxygen), enabling correct reaction center prediction. These results demonstrate that cross-attention provides a reliable inductive bias, enhancing both accuracy and interpretability.

### Case study: qualitative analysis

**Figure 4:**
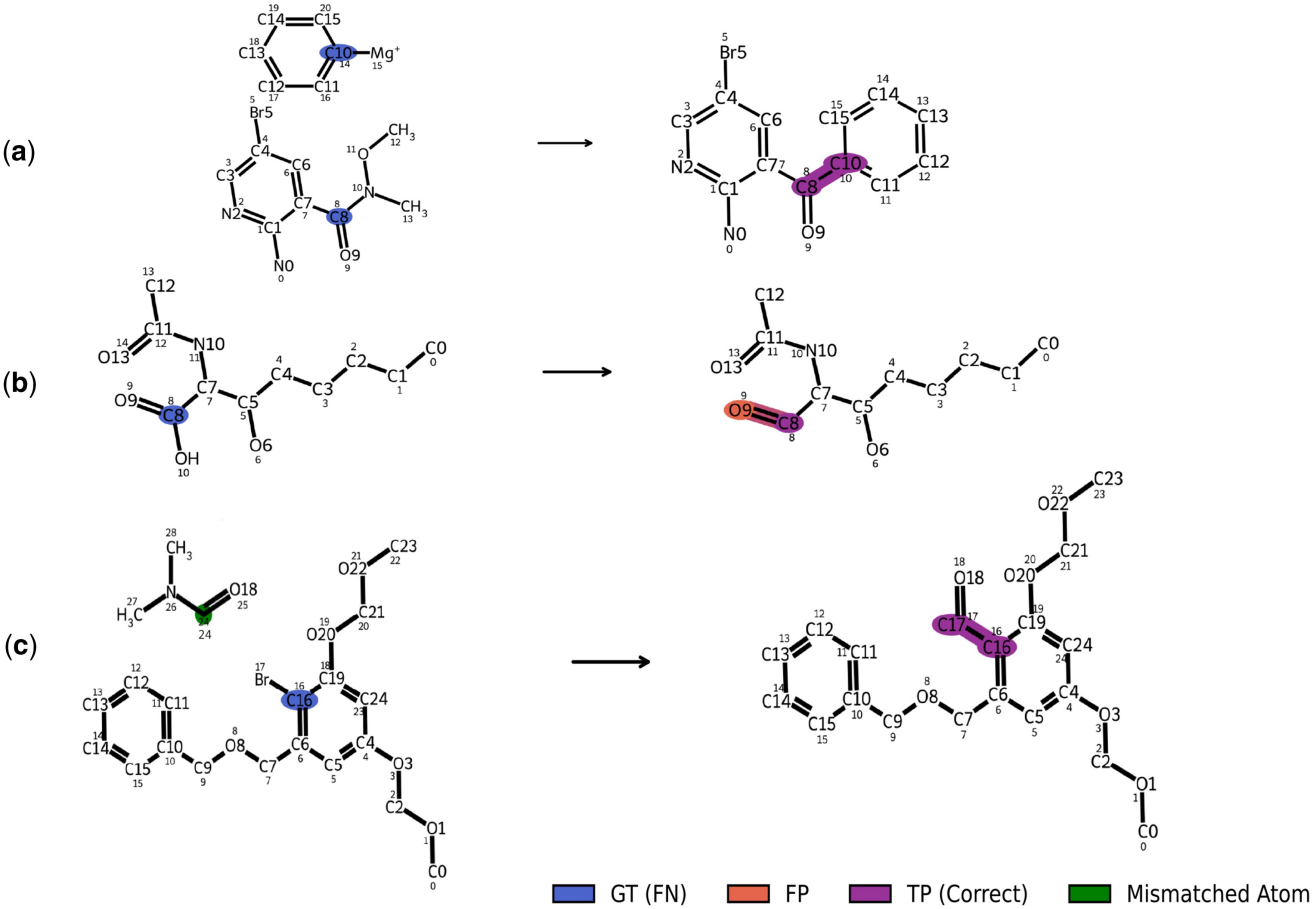
Representative MARCC predictions on USPTO-50K test set reactions. Each row shows a different reaction: (a) MARCC accurately predicts both atom mapping and reactivity. (b) MARCC correctly maps atoms but falsely classifies atom 9 as reactive (orange). (c) MARCC identifies the correct reaction center but misaligns atom 17 in the mapping (green).

Figure 4 illustrates three representative reactions from the USPTO-50K test set, highlighting MARCC’s behavior across diverse prediction scenarios.

In Fig. 4a, MARCC correctly predicts both atom mapping and the reaction center, including reactive bonds, reflecting the model’s ability to handle non-trivial rearrangements. In Fig. 4b, all atoms are mapped correctly, but the model falsely labels atom 9 (an oxygen) as reactive—likely due to overgeneralization from learned chemical priors. In Fig. 4c, the reaction center is correctly predicted, but one product atom is misaligned during atom mapping. Specifically, carbon atom 17 is incorrectly mapped to reactant atom 20 instead of the ground-truth atom 24. This discrepancy likely stems from limited structural context in a small molecule, where peripheral atoms may lack distinctive features.

To quantitatively validate our qualitative interpretability claims, we performed a structural analysis of the attention mechanism (Supplementary Sections S7–S8). Specifically, Section S7 provides a Multi-Head Attention analysis, while Section S8 offers fine-grained visualizations of reactant–product alignments. To ensure these findings generalize across the entire dataset, we conducted a systematic evaluation of prediction consistency (Section S6) and analyzed the statistical correlation between structural mapping and reaction center localization (Section S10).

## Conclusion and future work

We presented MARCC, a multi-task graph learning framework that unifies reaction center identification, reaction classification, and atom mapping within a single architecture. Its three main innovations—(i) mapping-guided cross-attention for reactant–product alignment, (ii) a dual-graph representation for bond-level reasoning, and (iii) imbalance-aware optimization—enable coherent reasoning across local and global aspects of molecular reactivity.

On the USPTO-50K benchmark, MARCC achieves state-of-the-art performance in reaction center localization and classification, while remaining competitive with dedicated atom-mapping systems. Ablation studies confirm that its multitask design enhances both accuracy and interpretability, making it well-suited for applications such as synthesis planning, reaction annotation, and mechanistic elucidation.

Beyond cheminformatics, MARCC offers a general design principle for reaction modeling: leveraging atom-to-atom alignment as a structural prior to unify local edits and global semantics. This paradigm holds promise for biochemical domains including enzymatic reactions, metabolic pathways, and drug metabolism. Future extensions will target retrosynthesis and multi-step synthesis planning, aiming to improve pathway inference and accelerate automated molecular discovery. In particular, we aim to enhance the model’s robustness to data scarcity and noise.

While the current work validates MARCC on a high-fidelity benchmark, the architecture is designed with inherent flexibility to scale toward more complex chemical spaces. Our framework naturally supports multi-product reactions by treating the product set as a disconnected graph, while the soft-attention mechanism in our cross-graph alignment remains robust to missing reagents by prioritizing existing structural correspondences. We intend to leverage these dataset-agnostic inductive biases to extend MARCC to larger, noisier corpora such as the Open Reaction Database (ORD) and USPTO-full. Future efforts will focus on utilizing the structural diversity of these datasets—combined with class-aware sampling and targeted data augmentation—to further enhance performance on rare transformations and complex multi-component systems. To further contextualize these advancements, future work will benchmark MARCC against architectures utilizing local environments and human-in-the-loop refinement, such as LocalMapper (Chen et al. 2024), exploring the interplay between fully automated global reasoning and expert-guided local mapping.

## Supplementary Material

btag193_Supplementary_Data

## Data Availability

Code and data are available at https://github.com/maryamastero/MARCC; an archived version is available via Zenodo (https://doi.org/10.5281/zenodo.18500230).
