# Supplementary material for: A cross-attentive multi-task graph learning framework for chemical reaction modeling
Source: Bioinformatics. 2026 Apr 20;42(5):btag193. doi: 10.1093/bioinformatics/btag193 (PMC13181183; doi:10.1093/bioinformatics/btag193)
Supplement: btag193_Supplementary_Data [file btag193_supplementary_data.pdf]

Supplementary Material for: A cross-attentive multi-task graph learning framework for chemical reaction modeling

Maryam Astero, Anchen Li, Elena Casiraghi, Juho Rousu

S1 Overview of the MARCC Workflow

Algorithm 1 MARCC: Mapping-Assisted Reaction Center and Classification

Require:
Reactant graph $G_R = (A_R, X_R, E_R)$,
Product graph $G_P = (A_P, X_P, E_P)$

Ensure: Atom mapping M, atom reactivity scores $\hat{Y}$, bond reactivity scores $\hat{Z}$, reaction class logits $\hat{C}$

1: **Graph Encoding:** Encode G_R and G_P with shared GINE encoder
$H_R \leftarrow \mathrm{GINE}(A_R, X_R, E_R)$
$H_P \leftarrow \mathrm{GINE}(A_P, X_P, E_P)$

2: **Atom Mapping (Auxiliary Task):**
$\hat{M} \leftarrow H_P H_R^\top$
$M \leftarrow \mathrm{Sinkhorn}(\hat{M})$

3: **Cross-Attention:**
$\tilde{H}_P \leftarrow \mathrm{Attention}(Q = H_P, K = MH_R, V = MH_R)$
$\hat{H}_P \leftarrow \mathrm{MLP}([H_P \parallel \tilde{H}_P])$

4: **Reaction Center Prediction:**
$\hat{Y} \leftarrow \mathrm{MLP}_{\mathrm{atom}}(\hat{H}_P)$
$\hat{Z}_{ij} \leftarrow \mathrm{MLP}_{\mathrm{bond}}([\hat{H}_P^{(i)} \parallel \hat{H}_P^{(j)} \parallel E_P^{(ij)} \parallel \hat{Y}_i, \hat{Y}_j])$

5: **Reaction Classification:**
$H_{\mathrm{graph}} \leftarrow \mathrm{mean\_pool}(\hat{H}_P)$
$\hat{C} \leftarrow \mathrm{MLP}_{\mathrm{class}}(H_{\mathrm{graph}})$

6: **Training Objective:**
$\mathcal{L}_{\mathrm{total}} \leftarrow \lambda_{\mathrm{amap}}\mathcal{L}_{\mathrm{amap}} + \lambda_{\mathrm{react}}(\mathcal{L}_{\mathrm{atom}} + \mathcal{L}_{\mathrm{bond}}) + \lambda_{\mathrm{rxn}}\mathcal{L}_{\mathrm{rxn}}$
where:
$\mathcal{L}_{\mathrm{amap}}$: Soft cross-entropy over M
$\mathcal{L}_{\mathrm{reac}}, \mathcal{L}_{\mathrm{bond}}$: Focal-Dice hybrid losses
$\mathcal{L}_{\mathrm{rxn}}$: Cross-entropy over $\hat{C}$

S2 Dual Graph Construction

Given a molecular graph $G = (V, E)$, we define its dual graph $G^D = (V^D, E^D)$ as follows:

- Each bond $e_{ij} \in E$ in the original graph becomes a node in the dual graph: $V^D = \{e_{ij} \mid (i, j) \in E\}$.
- Two nodes $e_{ij}, e_{jk} \in V^D$ are connected in G^D if they share an atom in G: $E^D = \{(e_{ij}, e_{jk}) \mid v_j \in V \text{ and } e_{ij}, e_{jk} \text{ incident on } v_j\}$.

Bond-level attributes from G are inherited as node features in G^D, while edge features in G^D are constructed from the atom embeddings of shared atoms. This design supports spatially localized bond reasoning for complex reaction center predictions.

An example of this dual graph construction is illustrated in Figure S1.

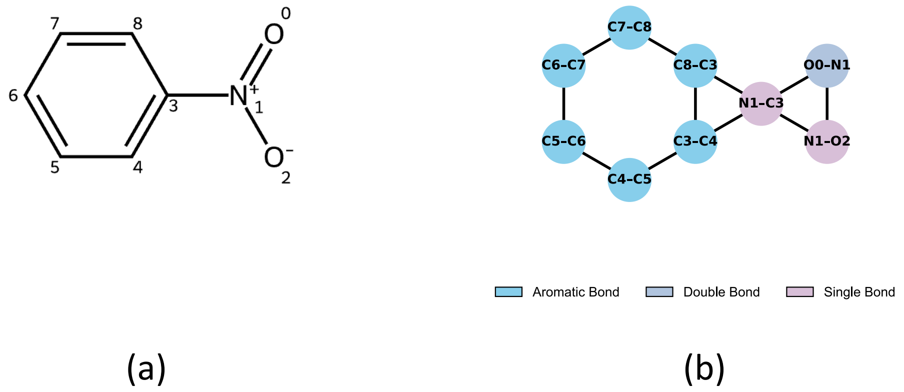

Figure S1: Illustration of dual graph construction. (a) shows a molecular graph where atoms are nodes and bonds are edges. (b) shows the corresponding dual graph, where each bond becomes a node, and edges are formed between bonds that share an atom in the original graph.

S3 Loss Formulation

In the USPTO-50K dataset, reactive sites are rare: only 6.54% of atoms and 3.17% of bonds are labeled as reactive. This pronounced class imbalance presents a major challenge for supervised learning, as conventional loss functions (e.g., cross-entropy) tend to bias the model toward the dominant negative class, leading to poor sensitivity and degraded recall on the minority class.

To address this, we adopt a hybrid loss strategy that combines Focal Loss and Dice Loss.

S3.1 Focal Loss.

To address class imbalance and focus learning on difficult examples in the reactivity prediction task, we employ a binary focal loss. Given a prediction probability $p_i \in (0, 1)$ and binary label $y_i \in \{0, 1\}$, the focal loss is:

$$\mathcal{L}_{\text{focal}} = -\alpha(1 - p_i)^{\gamma} y_i \log(p_i) - (1 - \alpha) p_i^{\gamma} (1 - y_i) \log(1 - p_i),$$

where $\gamma > 0$ controls the down-weighting of well-classified examples, and $\alpha \in [0, 1]$ adjusts the loss contribution of positive and negative classes.

S3.2 Dice Loss.

To encourage overlap between predicted and true labels, especially under imbalance, we incorporate a Dice loss. For a set of predicted probabilities $p = \{p_i\}$ and corresponding labels $y = \{y_i\}$, it is defined as:

$$\mathcal{L}_{\text{dice}} = 1 - \frac{2 \sum_i p_i y_i + \epsilon}{\sum_i p_i + \sum_i y_i + \epsilon},$$

where ϵ is a small constant to prevent division by zero.

S3.3 Adaptive Loss Combination.

We combine focal and Dice losses using a dynamically reweighted scheme to balance their gradient contributions. The final hybrid loss is computed as:

$$\mathcal{L}_{\text{hybrid}} = \lambda_{\text{dice}} \cdot \mathcal{L}_{\text{dice}} \cdot \left(\frac{\mathcal{L}_{\text{focal}} + \epsilon}{\mathcal{L}_{\text{dice}} + \epsilon} \right)^{1/2} + (1 - \lambda_{\text{dice}}) \cdot \mathcal{L}_{\text{focal}},$$

where $\lambda_{\text{dice}} \in [0, 1]$ determines the trade-off between Dice and focal components. This formulation adaptively adjusts the influence of each loss term to maintain stable optimization dynamics in the presence of extreme label sparsity.

S4 Atom and Bond Feature Specification

We detail the features extracted from RDKit used to construct molecular graph representations. All categorical features are one-hot encoded, and scalar features are scaled as described in the codebase.

S5 Hyperparameter Optimization Setup

Table S3 summarizes the Optuna search space, pruning strategies, and runtime constraints used in all hyperparameter optimization experiments.

Table S1: Atom features used in graph construction

**Feature Group**	**Description**	**Dim**
Atom type	One-hot over permitted list + 'NA'	17
Heavy neighbor count	{0, 1, 2, 3, 4, greater than 4}	6
Is in ring	Boolean flag	1
Is aromatic	Boolean flag	1
Number of Hs	{0, 1, 2, 3, 4, greater than 4}	6
Explicit valence	{1–6}	6
Implicit valence	{0–5}	6
Atomic mass (scaled)	Scalar	1
Van der Waals radius (scaled)	Scalar	1
Covalent radius (scaled)	Scalar	1
Hybridization	{S, SP, SP2, SP3, SP3D, SP3D2, OTHER}	7
Formal charge	{-3 to +3, Extreme}	8
Chirality	{Unspecified, CW, CCW, Other}	4
Hydrogen count	{0, 1, 2, 3, 4, greater than 4}	6
**Total**		**71**

Table S2: Bond features used in graph construction

**Feature Group**	**Description**	**Dim**
Bond type	{Single, Double, Triple, Aromatic}	4
Is conjugated	Boolean flag	1
Is in ring	Boolean flag	1
Is aromatic	Boolean flag	1
Ring size	{0–8}	9
Stereochemistry	{Z, E, Any, None}	4
**Total**		**20**

Table S3: Optuna hyperparameter optimization setup.

**Component**	**Configuration**
Trials per study	30
Pruning strategy	ASHA
Alternative pruner	Median
Per-trial wallclock limit	120 minutes
Early stopping	Patience = 10
**Search space**	
Embedding dimension	{256, 384, 512, 640, 768}
Edge hidden dimension	{256, 384, 512, 640}
Number of layers	[3, 7] (integer)
Attention heads	{2, 4, 8}
Learning rate	[5e-5, 3e-4] (log-uniform)
Weight decay	[1e-6, 3e-4] (log-uniform)
Task weights ($\lambda_{\text{amap}}, \lambda_{\text{atom}}, \lambda_{\text{bond}}, \lambda_{\text{reaction}}$)	[0.05, 0.6], normalized to 1
Dice loss weight	[0.2, 0.8]
Atom loss pos-weight	[2.0, 10.0]
Bond loss pos-weight	[2.0, 15.0]
Batch size	{16, 24, 32, 48}
Cosine annealing T_0	{25, 50, 100}
Cosine annealing η_{min}	[1e-7, 1e-5] (log-uniform)

S6 Prediction Consistency Between Local and Global Tasks

To evaluate internal consistency between MARCC's predictions, we compute the percentage of test set reactions for which both the predicted reaction class is correct and the predicted Top-1 edit set exactly matches the ground truth.

As shown in Figure S2, MARCC achieves a mean consistency rate above 96%, with several reaction classes (e.g., 0, 1, 5, 9) reaching near-perfect agreement. Classes such as 3 and 8 exhibit lower consistency, likely due to edit ambiguity or structural diversity. This alignment indicates that the model's global classification decisions are supported by chemically plausible local edits.

S7 Multi-Head Attention Analysis

To provide deeper insight into MARCC's interpretability, we visualize per-head attention patterns for the same reaction presented in Figure 3a. The goal is to understand how each attention head contributes to identifying product–reactant correspondences, especially at reactive centers.

In the heatmaps, each panel corresponds to one of the eight attention heads. The horizontal axis represents reactant atoms, and the vertical axis represents product atoms. The intensity of each cell indicates the attention score from a

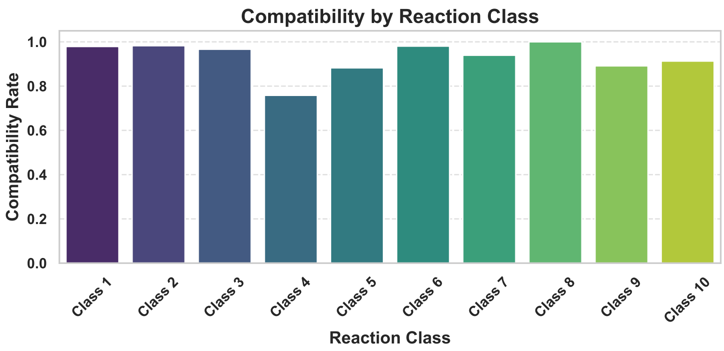

Figure S2: Consistency between predicted reaction classes and Top-1 edit sets across reaction classes in USPTO-50K. A high score indicates that global transformation predictions are structurally supported by correct mechanistic edits.

given product atom to a specific reactant atom.

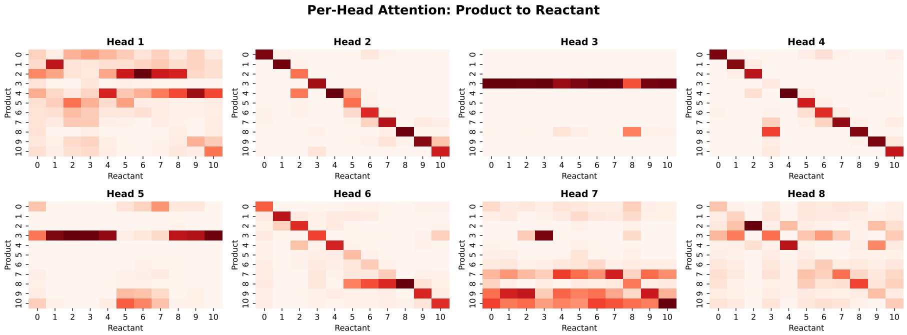

Figure S3: Multi-head cross-attention heatmaps from product to reactant atoms for the reaction in Figure 3a. Each subplot visualizes the attention weights from one of the eight attention heads in the MARCC model for a representative reaction. Darker shades indicate stronger attention between a product atom (row) and a reactant atom (column). The diversity across heads reveals complementary alignment behaviors—some attend to directly mapped atoms (diagonal bands), while others capture contextual or diffuse structure, enhancing robustness.

Key observations:

- Head 3 and Head 5 show highly focused attention on a specific row (product atom), concentrating most weight on a single reactant atom—this corresponds to a strong match at the reaction center.
- Heads 2, 4, and 6 exhibit near-diagonal attention patterns, reflecting approximate one-to-one mappings that capture global atom-level alignment.

- Head 8 displays more dispersed attention across multiple reactant atoms, suggesting its role in modeling broader contextual dependencies or secondary interactions.
- Head 1 and Head 7 reveal product-to-reactant interactions away from the diagonal, which may help resolve asymmetric mappings or support ambiguous transformations.

This diversity across attention heads provides a mechanism for robust alignment even in structurally ambiguous or symmetric scenarios.

S8 Fine-Grained Attention Visualization

To support interpretability, we analyze the attention behavior of MARCC at a fine-grained level. Figure S4 shows the per-product-atom attention distribution over reactant atoms for the same example discussed in the main paper (Figure 3a).

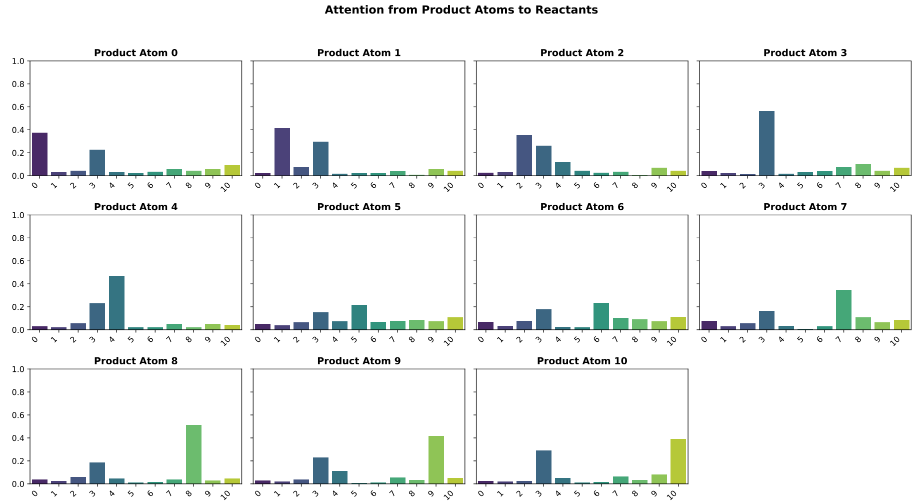

Figure S4: Single-product-atom attention distributions over reactants (example from Figure 3a). Each subplot shows the attention weights from one product atom to all reactant atoms. Bar height indicates the relative attention score. High attention is focused on chemically aligned atoms—especially reactive centers—demonstrating MARCC's ability to localize structural correspondence through soft attention. Colors indicate atom indices for visual separation and carry no semantic meaning.

The attention distribution plots in Figure S4 illustrate how MARCC's cross-attention mechanism facilitates structural alignment between the product and reactant graphs. Each subplot displays the normalized attention weights for

a specific product atom across all candidate reactant atoms, providing a soft alignment signal used to enrich product embeddings with reactant-side context.

Attention is typically peaked for both reactive and non-reactive atoms, as the mechanism primarily captures the structural correspondence between the two sides of the reaction. For instance, the true reaction center (e.g., Product Atom 3) concentrates most of its mass on a single reactant atom, consistent with a stable correspondence.

In contrast, some atoms (e.g., Product Atom 4) display bimodal attention with two prominent reactant candidates. This behavior is expected when multiple reactant atoms reside in very similar chemical environments (e.g., local symmetry), or when the model incorporates nearby structural context to support downstream reactivity prediction.

These visualizations validate that the cross-attention module functions as a robust alignment layer, ensuring that product atoms are correctly contextualized by their reactant-side origins.

S9 Ablation Study of Dual-Graph and Attention Components

To quantify the individual contributions of guided cross-attention and dual-graph features, we perform an ablation study across four MARCC variants (Table S4): (i) the full MARCC model with both components enabled; (ii) a variant without the dual-graph module while retaining guided attention; (iii) a variant without attention but preserving dual-graph reasoning; and (iv) a minimal baseline in which both components are removed. Each variant is evaluated using symmetry-aware atom mapping accuracy, edit accuracy ($n = 1$), and classification accuracy.

Table S4: Ablation study of MARCC variants. Bold indicates the best performance for each metric (higher is better).

Model Variant	Edit Acc. (%)	Class Acc. (%)	Mapping Acc. (%)
MARCC (Full)	**99.1**	**97.2**	**98.2**
– Dual Graph	85.4	96.1	98.0
– Attention	67.2	76.7	97.9
– Attention, – Dual	66.6	75.9	97.1

The ablation results highlight that while atom-mapping accuracy remains relatively stable across variants, the Edit Accuracy is highly sensitive to the structural representation. The sharp decline observed upon removing the dual-graph module (Variant ii) indicates that bond-centric reasoning is the primary driver for identifying reactive centers. This confirms that treating bonds as first-class nodes allows the GINE encoder to more effectively learn the 'edits' (bond formations and breakages) that define a chemical transformation, rather

than relying solely on atom-level neighbor aggregation.

S10 Statistical Dependency between Mapping and Edit Accuracy

To evaluate how structural alignment influences chemical reasoning, we analyzed the synergy between atom mapping accuracy and reaction center (RC) identification success. Given the binary nature of instance-level RC accuracy (where a prediction is either exactly correct or incorrect), we examined whether successful RC localization is statistically dependent on the quality of the underlying structural mapping.

As illustrated in Figure S5a, there is a distinct disparity between the mapping quality of successful and failed predictions. For reactions where the model correctly identified the reaction center, the atom mapping accuracy is concentrated at 1.0. Conversely, failures in RC localization correlate with a visible shift toward lower mapping accuracy and higher variance.

To quantify this observation, we performed a Mann-Whitney U test, which confirmed that the atom mapping accuracy for correct RC predictions is statistically superior to that of incorrect ones ($p = 1.48 \times 10^{-29}$). Furthermore, the class-specific breakdown in Figure S5b confirms that this structural-predictive coupling is a fundamental trait of the MARCC architecture, persisting across both high-frequency and rare transformation types.

As shown in Table S5, this architectural robustness allows MARCC to maintain high predictive fidelity even in highly skewed classes. Notably, rare classes such as Protections (5), Reductions (8), and Others (10) achieve a 100% mean edit accuracy, grounded in high-precision structural correspondences.

Table S5: Quantitative Performance Breakdown by Reaction Class. Mean accuracies highlight the model's robustness across both high-frequency and rare transformation types on the USPTO-50K test set.

**Reaction Class (ID)**	**Sample Size**	**Amap Acc**	**Edit Acc**
Heteroatom Alkylation (1)	1516	0.981	0.993
Acylation (2)	1190	0.986	0.997
C-C Bond Formation (3)	567	0.969	0.993
Heterocycle Formation (4)	91	0.927	0.956
Protections (5)	68	0.990	1.000
Deprotections (6)	824	0.991	0.995
Oxidations (7)	462	0.987	0.983
Reductions (8)	82	0.992	1.000
Functional Group Conv. (9)	184	0.972	0.946
Others (10)	23	0.968	1.000
**Global Total / Average**	**5007**	**0.982**	**0.991**

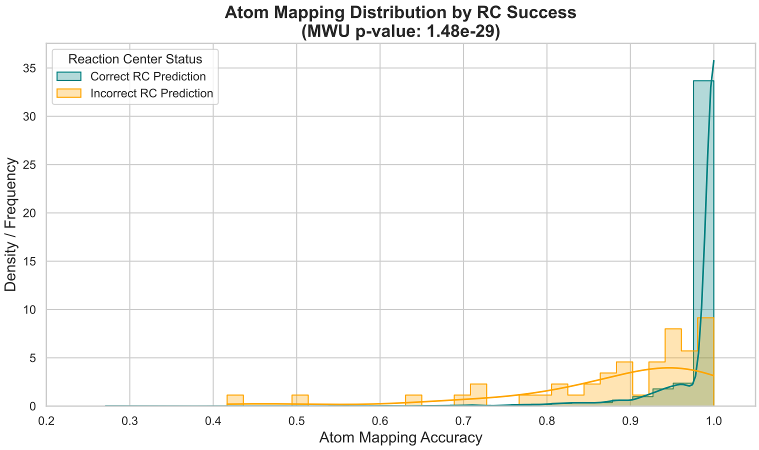

(a) Global Distribution Overlay

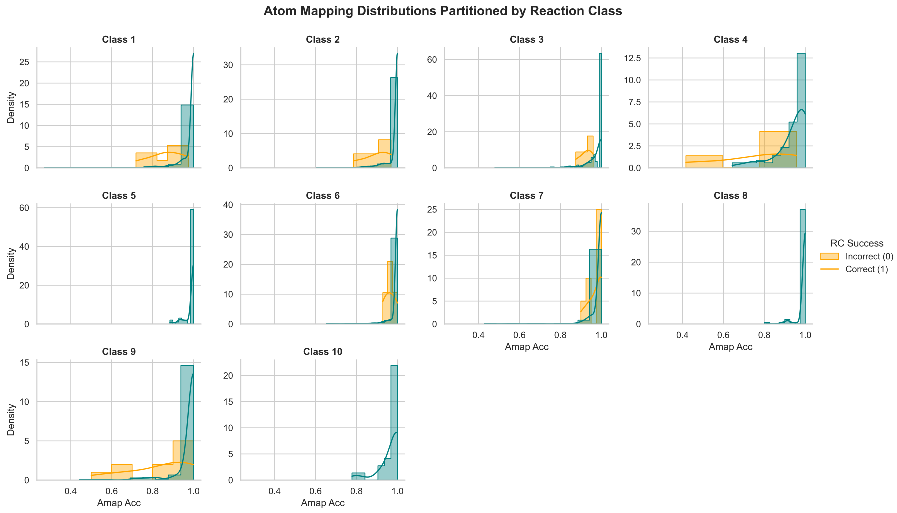

(b) Class-Specific Distributions

Figure S5: Distribution of Atom Mapping Accuracy partitioned by RC Prediction Success. (a) Global overlaid histograms and Kernel Density Estimation (KDE) curves illustrate that successful RC predictions (teal) are characterized by a sharp peak at 1.0 mapping accuracy, while failed predictions (orange) exhibit a lower-accuracy distribution. (b) Faceted histograms demonstrate that this dependency is universal across all ten reaction classes.
